# Continuous Membrane-Based Screening System for Biocatalysis

**DOI:** 10.3390/membranes1010070

**Published:** 2011-02-25

**Authors:** Evgenij Lyagin, Anja Drews, Subhamoy Bhattacharya, Matthias Kraume

**Affiliations:** 1 Chair of Chemical and Process Engineering, Technische Universität Berlin, Sekr. MA 5-7, Straße des 17. Juni 135, 10623 Berlin, Germany; E-Mails: subhamoy@mailbox.tu-berlin.de (S.B.); matthias.kraume@tu-berlin.de (M.K.); 2 HTW Berlin, Department of Engineering II, School of Life Science Engineering, Wilhelminenhofstr. 75a, 12459 Berlin, Germany; E-Mail: anja.drews@htw-berlin.de; 3 Institute for Chemistry, Department of Enzyme Technology, Technische Universität Berlin, Sekr. TC 4, Straße des 17. Juni 124, 10623 Berlin, Germany

**Keywords:** screening system, enzyme membrane reactor, membrane continuous stirred tank reactor, cellulose hydrolysis, fouling control

## Abstract

The use of membrane reactors for enzymatic and co-factor regenerating reactions offers versatile advantages such as higher conversion rates and space-time-yields and is therefore often applied in industry. However, currently available screening and kinetics characterization systems are based on batch and fed-batch operated reactors and were developed for whole cell biotransformations rather than for enzymatic catalysis. Therefore, the data obtained from such systems has only limited transferability for continuous membrane reactors. The aim of this study is to evaluate and to improve a novel screening and characterization system based on the membrane reactor concept using the enzymatic hydrolysis of cellulose as a model reaction. Important aspects for the applicability of the developed system such as long-term stability and reproducibility of continuous experiments were very high. The concept used for flow control and fouling suppression allowed control of the residence time with a high degree of precision (±1% accuracy) in a long-term study (>100 h).

## 1. Introduction

Enzyme membrane reactors have been shown to be suitable for different types of reactions such as hydrolysis of macromolecules, cofactor-regenerating reactions, hydrolysis catalyzed by lipases or reactions in reverse micelles [1]. Furthermore, there are numerous examples of industrial tasks of enzymatic membrane reactors including the resolution of N-Acetyl-amino acids [2], production of *(S)*-*tert.*-leucine and *(S)*-neopentyl-glycine or L-ornithine and its salts [3]. For the successful design of pilot-scale and subsequently full-scale plants, typically lab-scale systems for enzyme, substrate and condition screening are used. Due to the fact that currently available screening systems can mainly only be operated in batch or fed-batch mode, the screening results achieved in such conditions can be misleading for membrane reactor application and operation. To the best of our knowledge, parallelized membrane-based small scale reactors are not available yet. In contrast to industrial systems, this small scale presents different challenges which have to be overcome in the design of such a screening and characterization system: precise control of flow rates at such small values, parallelism, characterization and scalability of power input, *etc.* This study thus aims to evaluate the first results achieved from the novel membrane-based screening system developed in our previous study [4] in terms of long-term stability, fouling control, parallelism of reactors and reproducibility of results. The main features of the developed system are:

- Possibility of continuous substrate feeding and continuous product removal;
- Suitability for homogeneous catalysis (non-immobilized enzymes can be used, since mass transfer limitations are smaller in comparison to the use of immobilized enzymes);
- Monitoring and control of temperature, pH, residence time and power input;
- Possibility of parallel operation (initially two reactors in parallel have been implemented);
- Low price.

## 2. Materials and Methods

### 2.1. Screening System

**Figure 1:**
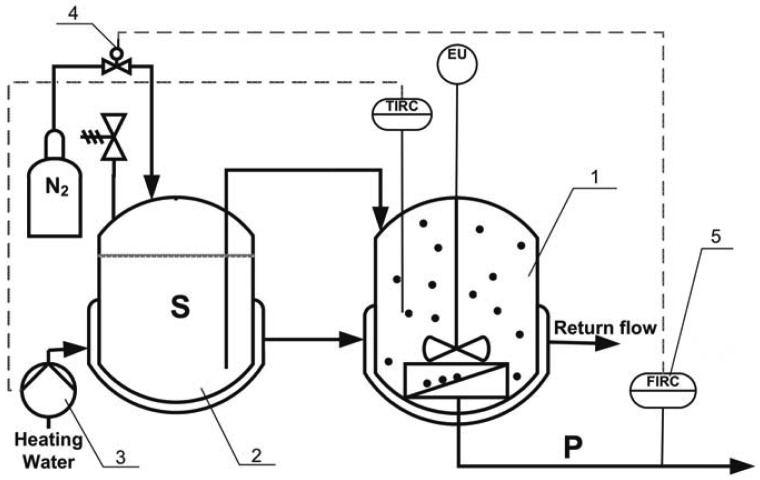
Developed screening system based on the membrane continuous stirred tank reactor (MCSTR) concept: (1) membrane reactor; (2) substrate tank; (3) thermostat; (4) pressure regulator; (5) precision balance;● enzymes; S: substrate; P: product [4].

The main components of the developed screening system, shown in Figure 1, are: the membrane reactor (modified XFUF-047 dead-end test cell, 90 mL volume, up to 14.7 cm^2^ eff. membrane surface area, *Millipore Corporation, USA*), a mixing device (MIX 1, *2 MAG, Germany*), a thermostat (D1, *Thermo Haake GmbH, Germany*), a pressure regulator (MPPE-3, *Festo AG, Germany*) and an electronic precision balance (ALT 310, *Kern & Sohn GmbH, Germany*) to monitor the permeate flow rate. The data from the balance were logged on a computer with a frequency of 4 Hz and used for flow rate control. *Visual Designer*™-Software (Version 4.0) was used to modulate and optimize the proportional integral (PI) controller settings.

### 2.2. Choice of Model Reaction, Substrate, Enzyme and Membrane Material

**Figure 2:**
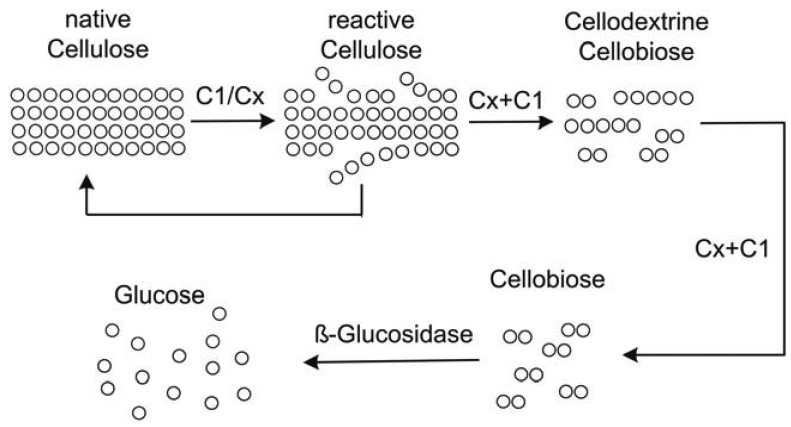
Suggested reaction mechanism of cellulose hydrolysis with a cellulase complex; C_1_: Exocellulase; C_X_: Endocellulase (adapted from [7]).

For the proof of concept of a novel reactor set-up, it should be operated with a known model reaction. Here, the hydrolysis of cellulose was chosen as a model reaction because of its significance in the energy sector, and food and chemical industries [5] and because it is strongly inhibited by its products glucose and cellobiose, which can only be prevented by *in situ* product removal [6]. Furthermore, this reaction cannot be performed with immobilized enzymes since for an adequate enzyme-substrate contact either substrates or enzymes should be dissolved. Figure 2 shows the suggested reaction mechanism of cellulose hydrolysis.

*α*-*Cellulose* (C8002, Sigma-Aldrich Corporation, USA) was chosen as the substrate. The enzyme used was cellulase from *Trichoderma reesei* (C8546, Sigma-Aldrich Corporation, USA), with a molecular weight range of 48–52 kDa [8]. The membrane chosen was an ultrafiltration membrane with 10 kDa molecular weight cut off (MWCO) (UP010, PES, Microdyn-Nadir GmbH, Germany) and was assumed to be impermeable for the enzyme used. This assumption is based on the fact that even a membrane with a higher MWCO of 30 kDa (PM30, PES, Amicon, Millipore Corporation, USA) was shown to retain around 90% of β-*Xylanase* from *Trichoderma reesei* (MW 20 kDa, [9]). At the same time, it is clear that products with MWs of 180 and 342 Da will not be retained by the chosen membrane.

### 2.3. Analytical Methods

The total concentration of both products, glucose and cellobiose, was measured using refractometry (DD-7, ATAGO Co., Ltd.). The cellulose conversion was calculated from the product concentration measured in permeate samples.

### 2.4. Enzyme Activity Test

Enzyme concentration is typically given in terms of activity. One enzyme activity unit is defined as the amount of enzyme that liberates 1 μmol/h of glucose from Sigmacell^®^-cellulose (S3504, 20 μm particle size) at T = 37 °C and pH = 5 over a period of 2 h. The enzyme activity test was performed in accordance with the *Sigma-Aldrich Control Test Procedure* [10].

### 2.5. Operation Conditions

Flux was set to J = 15–60 L/(m^2^·h) depending on the desired residence time (τ =12–3 h). The stirrer speed was varied from 100–750 rpm. As a reference, operating conditions from a previous study were used [4]: τ = 6 h, c_E_ = 1120 U/L, c_S,0_ = 25 g/L, T = 40 °C, pH = 4.66 (sodium acetate buffer).

## 3. Results and Discussion

### 3.1. Residence Time Control and Fouling Suppression

**Figure 3:**
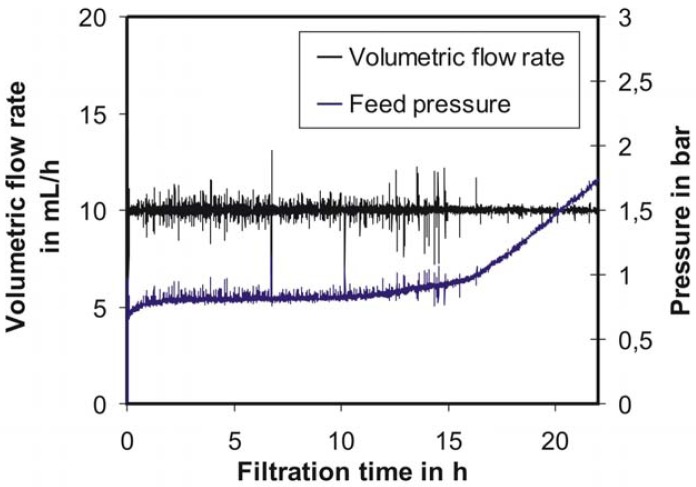
Volumetric flow rate and feed pressure development over time, c_S,0_ = 25 g/L, T = 40 °C [4].

Residence time, *i.e.*, flow rate control, was realized with the feed pressure as the actuating variable. The system responds to a step pressure change with a PT_n_-behavior. Thus, the disturbances occurring in such systems can be balanced with a closed-loop control and a PI/PID controller. The process of determining the optimal PI/PID settings was described previously [4]. Figure 3 presents typical feed pressure development in the membrane reactor over time. With an implemented PI controller and corresponding settings, the desired flow, and thus the desired residence time (e.g., 10 mL/h and 9 h, respectively) could be precisely controlled over a long period. The volumetric flow rate was 9.998 mL/h on average over 20 h and the standard deviation 0.228 mL/h (calculation based on the assumption of a Gaussian distribution of the data shown in Figure 3). The flux fluctuations can be attributed to degassing of N_2_ from the permeate because of the pressure gradient across the membrane. The pressure fluctuations are produced by the PI controller and compensate the volumetric flow rate. The pressure increase after approximately 16–17 h can be explained by membrane fouling (typical 3-stage transmembrane pressure (TMP) behavior in constant flux operation [11]). Although the implemented PI controller shows the ability to compensate even such a high permeability decline, the system itself shows an inner instability. For example, the pressure with the gradient shown in Figure 3 will reach the maximal acceptable value for the membrane reactor (about 5.5 bar) after approximately 50 h. In this case, the safety valve will be activated and the experiment will be stopped. Thus, for long-term studies, a concept for fouling suppression was worked out based on membrane and deposit relaxation. The typically applied membrane back flush is neither possible nor desirable here as it would mean a loss and re-introduction of permeate.

**Figure 4:**
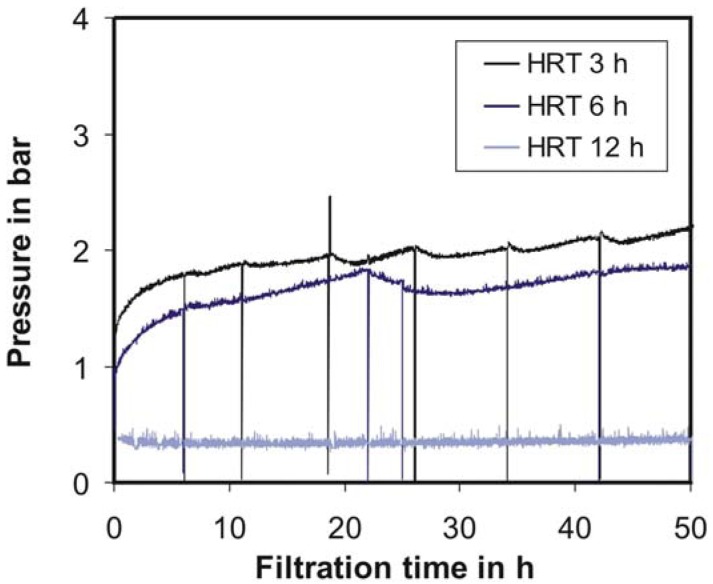
Fouling suppression during continuous cellulose conversion with different hydraulic residence times (HRT) from 3 to 12 h; c_E_ = 1120 U/L, c_S,0_ = 25 g/L, T = 40 °C, pH = 4.66, n = 100 min^−1^.

Figure 4 shows the results of this simple concept for fouling suppression. Thereby, after approximately every 6–8 up to 20–25 h (depending on the residence time) the PI controller is deactivated and the pressure released. Since the membrane is not pressurized at this time, the deposit layer formed on the membrane surface can be removed easier by means of the mixing device. After 1–2 min, the PI controller will be activated again. A rather rapid pressure increase can be observed at the beginning (0–3 h). However, this lasts longer than the typically observed conditioning phase [11]. In a second phase (Figure 4, from 3 to 6 h; Figure 3, from 3 to 15 h), the pressure gradient is much smaller than in the first phase. Starting from 6 h, each pressure release led to a pressure drop, which achieves a local maximum after 2–3 h. After these local pressure minima, an increase of pressure was always detected. Admittedly this slow pressure drop after a pressure release was surprising, because a much faster decrease was expected. Therefore, the reproducibility of this effect points to a special type of substrate-membrane interaction. With the described flow control and fouling suppression concepts, the desired residence time could be controlled with an accuracy of ±1% (data not shown).

### 3.2. Cellulose Conversion Experiments

#### 3.2.1. General

All experiments were carried out in parallel. The graphs shown are averaged values from two parallel experiments and the error bars represent the actual values from these experiments. The conversion in batch operation was calculated under the assumption of an ideally mixed reactor (Equation 1):

(1) $${\text{Conversion}}_{\text{Batch}}(t)=\frac{c_{\text{Sample}}(t)}{c_{S,0}}\cdot 100\%$$

In continuous operation, the insoluble substrate and enzymes were added to the reactor only at the beginning. Buffer was added continuously and product solution withdrawn at the same rate during the entire reaction period. The conversion was calculated also under the assumption of an ideally mixed reactor (Equation 2):

(2) $${\text{Conversion}}_{\text{Continuously}}(t)=\frac{V_{\text{Collected}}(t)\cdot \bar{c}(t)+V_{\text{Sample}}\cdot c_{\text{Sample}}(t)+V_{\text{Reactor}}\cdot c_{\text{Sample}}(t)}{V_{\text{Reactor}}\cdot c_{S,0}}\cdot 100\%$$

#### 3.2.1. Parallel Continuous and Batch Operations

**Figure 5:**
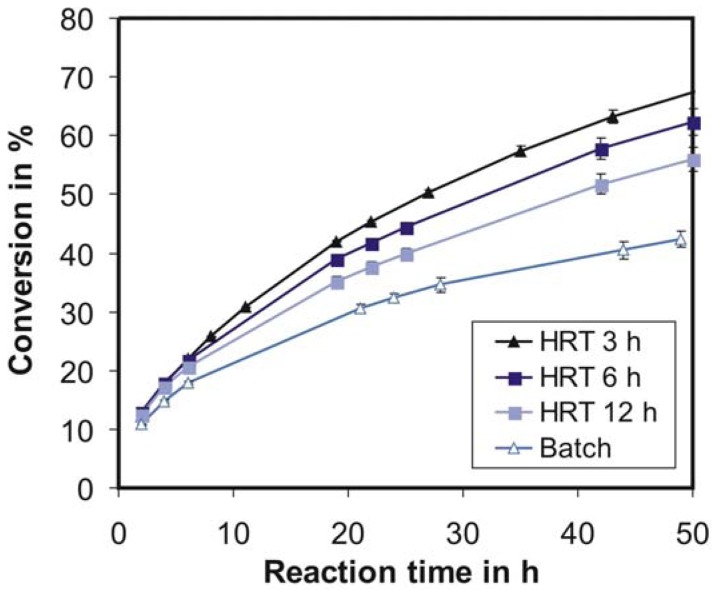
Cellulose conversion during parallel batch and continuous operations with different hydraulic residence times (HRT) from 3 to 12 h; c_E_ = 1120 U/L, c_S,0_ = 25 g/L, T = 40 °C, pH = 4.66, n = 100 min^−1^.

Figure 5 shows the comparison of cellulose conversion in batch and continuous mode under equivalent conditions. The continuous operation can increase the cellulose conversion by around 60% in comparison to batch (HRT 3 h *vs.* batch, both after 50 h reaction time). The reasons for the decrease of the reaction rate over time are different in batch and continuous mode. While in batch operation mode it is mainly due to the inhibition by the produced glucose and cellobiose, in case of continuous operation at short residence time it is rather the substrate depletion.

**Figure 6:**
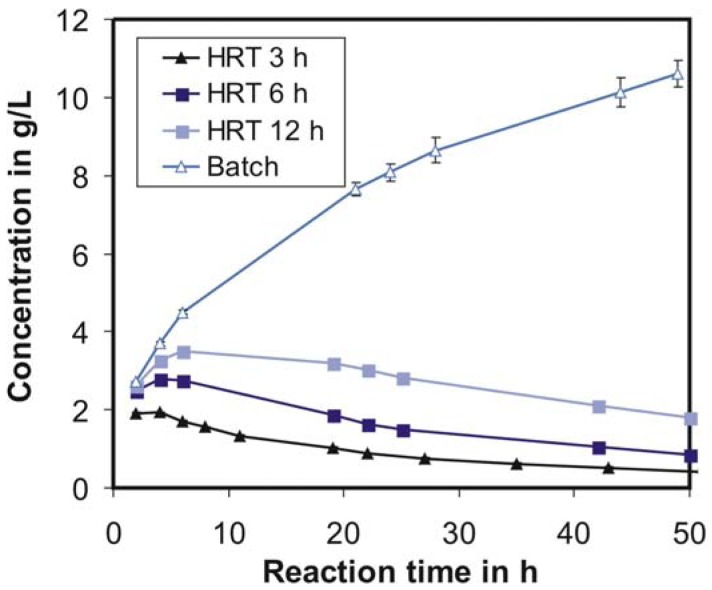
Product concentration profiles during cellulose conversion: comparison of parallel batch and continuous operations with different hydraulic residence times (HRT) from 3–12 h; c_E_ = 1120 U/L, c_S,0_ = 25 g/L, T = 40 °C, pH = 4.66, n = 100 min^−1^.

Figure 6 shows the corresponding product concentration profiles of the cellulose conversion shown in Figure 5. The product concentration during batch conversion increases over time, whereas in the case of a continuous operation an initial increase of product concentration is followed by a decrease. It is obvious that the produced sugars inhibit the enzyme, and this inhibition clearly increases with an increase in residence time. Therefore, for optimal process design it might be beneficial to operate the continuous process at variable HRTs (short in the initial phase and then increasing) so as to maintain a constant desirable product concentration with an allowable conversion rate.

**Figure 7:**
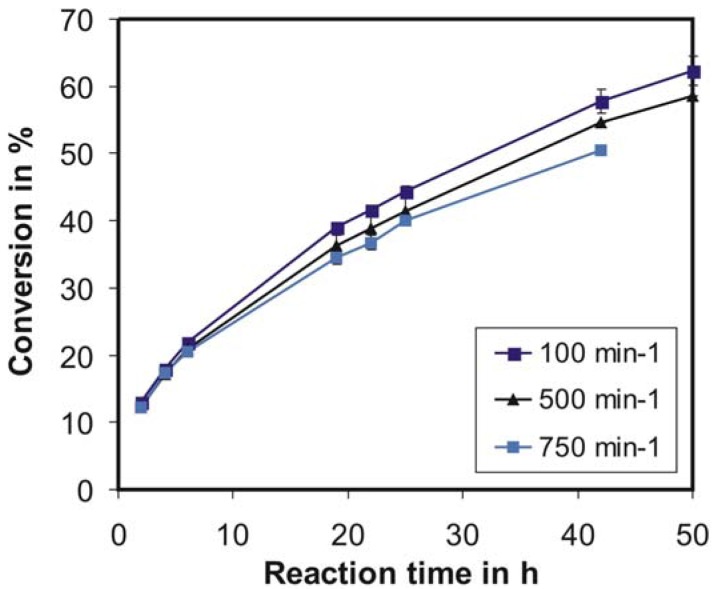
Cellulose conversion during parallel continuous operations for different stirrer speeds. c_E_ = 1120 U/L, c_S,0_ = 25 g/L, τ = 6 h, T = 40 °C, pH = 4.66.

Figure 7 shows the dependency of cellulose conversion on the stirrer speed. From the curves it can be concluded that the assumption of an ideally mixed reactor at 100 revolutions per minute (Section 3.2.1) was not so far from reality, because a subsequent increase of the rotational speed does not lead to an increase in cellulose conversion. This increase was expected with regard to reduction of the overall product concentration gradients. Also, an increase of the rotational speed should reduce the stagnant layer thickness on the substrate surface leading to smaller concentration gradients. With an increase in stirrer speed conversion losses were observed. Thus, enzyme deactivation plays a more dominant role compared to improvement in mass transfer due to increased stirrer speed, with respect to substrate conversion rates. Consequently, slower stirring was chosen and despite the fouling possibilities the flux could still be controlled at the desired value for 100 h (data not shown).

#### 3.2.2. Error Analysis of Parallel Continuous Operations

The comparison of the expected and actual errors was carried out for the reference conditions (see Section 2.5). The estimation of the expected error includes the following possible errors: enzyme, substrate and sample weighing, HRT-error, measurement error from refractometer and temperature error. The influence of substrate and sample weighing errors can be neglected because a precision balance was used. The maximal HRT-error between the parallel experiments (at reference conditions) shown in Figure 5 was ±0.1%. From the data shown in Figure 5, this results in an error of approximately ±0.01% of conversion. The precision of enzyme weighing was ±0.2 mg, which was ±1.1% of the total enzyme amount typically used. The error attributable to this was calculated as ±0.46% of conversion (calculation data not shown). The precision of the refractometer used is given by the manufacturer as ±0.005% [Brix], *i.e.*, ±0.05 g/L, and this generates a conversion error of ±1.85%. The temperature variation of ±0.2 °C causes a conversion error of ±0.1% (calculation data not shown). Thus, the overall expected error is approximately ±2.4% of conversion. The actual error in experiments at reference conditions (see Figure 5) is ±2.2%, which is very close to the expected error. This fact allows us to state that the developed system is suitable for carrying out high precision experiments.

## 4. Conclusions

The potential of a new continuous enzyme screening and characterization system was shown using the enzymatic hydrolysis of cellulose as a model reaction. This system is product inhibited and has a high fouling potential. Both limitations could be overcome by the developed reactor system. For a precise flow control (and, respectively, residence time) a PI/PID controller was realized. Furthermore, a concept for fouling suppression was developed. The concept used for flow control and fouling suppression allows control of the residence time with a high degree of precision (±1% accuracy) in a long-term study (>100 h, data not shown). Parallel experiments with variation of main process variables like residence time and power input were successfully carried out. Reproducible and comprehensible results between respective parallel experiments as well as experiments carried out under different conditions were achieved. The presented results show that the developed milliliter scale membrane reactor system is superior to traditional batch screening and characterization systems.

## List of Abbreviations

HRT: Hydraulic Residence Time
MCSTR: Membrane Continuous Stirred Tank Reactor
MWCO: Molecular Weight Cut Off
PI/PID: Proportional-Integral/ Proportional-Integral-Differential
TMP: Transmembrane Pressure
